# Skin γδ T Cells and Their Function in Wound Healing

**DOI:** 10.3389/fimmu.2022.875076

**Published:** 2022-04-11

**Authors:** Wengang Hu, Ruoyu Shang, Jiacai Yang, Cheng Chen, Zhihui Liu, Guangping Liang, Weifeng He, Gaoxing Luo

**Affiliations:** ^1^State Key Laboratory of Trauma, Burn and Combined Injury, Institute of Burn Research, Southwest Hospital, Third Military Medical University (Army Medical University), Chongqing, China; ^2^Chongqing Key Laboratory for Disease Proteomics, Chongqing, China

**Keywords:** γδT cells, wound healing, DETCs, Vγ4, Vγ6, homeostasis

## Abstract

For the skin immune system, γδ T cells are important components, which help in defensing against damage and infection of skin. Compared to the conventional αβ T cells, γδ T cells have their own differentiation, development and activation characteristics. In adult mice, dendritic epidermal T cells (DETCs), Vγ4 and Vγ6 γδ T cells are the main subsets of skin, the coordination and interaction among them play a crucial role in wound repair. To get a clear overview of γδ T cells, this review synopsizes their derivation, development, colonization and activation, and focuses their function in acute and chronic wound healing, as well as the underlining mechanism. The aim of this paper is to provide cues for the study of human epidermal γδ T cells and the potential treatment for skin rehabilitation.

## Introduction

γδ T cells (according to their γδ TCR) were first identified as a novel T-cell subset in the mid-1980s (1). As a gap between innate and adaptive immune response, they participate in regulating carcinoma (2), maintaining antimicrobial barrier (3), wound healing (4), psoriasis (5) and graft rejection (6). γδ T cells represent less than 5% of peripheral lymphocyte population in mice, human and rat (7, 8), whereas it constitutes a relatively large fraction of T lymphocytes in chicken, sheep, cattle and pig (15–50%) (8). In adult mice, γδ T cells are unequally distributed (9); there are less than 5% of total T cells in the lung, approximately 20–40% of the intraepithelial T cells of intestinal, approximately 10–20% of total T cells in the reproductive tracks, approximately 50–70% of skin dermal T cells and approximately 95% of epidermal T cells. In addition, they are divided into Vγ1-7 γδ T subsets according to the γ chain (10). Almost all γδ T cells in epidermis are dendritic epidermal T cells (DETCs: named by its dendritic morphology), expressing an invariant Vγ5Vδ1 TCR (according to Tonegawa’s nomenclature, which is adopted in this paper), equal to Vγ3Vδ1 TCR (according to Garman’s nomenclature) (11, 12). They maintain a homeostatic population by self-renew and can secrete growth factors such as IGF-1 (Insulin-like growth factor 1) and KGF-1/KGF-2 (keratinocyte growth factor 1/2) etc. (13) Most γδ T cells in dermis are Vγ4 T and Vγ6 Cells, they can secrete IL-17A (interleukin-17A), IFN-γ (interferon-γ) and the growth factors (4).

In humans, γδ T cells are classified based on the presented Vδ gene segment. Until now, there exists three true Vδ genes: Vδ1-3; and seven functional Vγ gene segments: Vγ2-5, Vγ8, Vγ9, and Vγ11 (14). Vδ1 γδ T cells primarily colonized in the dermis, and a small population is distributed in the epidermis, whereas Vδ2 TCRs are mainly distributed in peripheral blood and dermal (15, 16). Human epidermal γδ T cells play a functionally similar role as DETCs in promoting wound healing *via* secreting insulin-like growth factor 1 (IGF-1) and regulating cutaneous carcinoma (17, 18). However, they are not called DETCs as they do not possess dendritic morphology and take different molecular mechanisms in epidermis homing, antigen recognition and activation.

The skin, which is essential in defencing against external pathogens and environmental factors such as the microbes attack, ultraviolet radiation and heat injury (15, 19), serves as the largest interface between the body and the external environment. On one side, skin needs enough defending power to maintain homeostasis; on the other side, it needs fast and effective responses to repair the injury and restore the integrity upon injury or inflammation. Wound repair mainly contains four overlapping stages, which includes hemostasis, inflammation, proliferation and remodeling (20). Immune cells manage wound repair by secreting cytokines and chemokines to induce inflammatory microenvironment and promote re-epithelialization. DETCs, Vγ4 T cells and Vγ6 T cells are the main subsets of skin T lymphocytes and the equilibrium, coordination and interaction among them significantly affect their effectiveness in wound repair. This review primarily focuses on the discussion the rodent and murine γδ T cells, including their development, differentiation, colonization, activation, their functions and the underlining mechanism in wound healing. In addition, by consolidating the recent research breakthrough in the field, perhaps this article may also provide potential cues for the study of human skin γδ T cells and the potential treatment for skin rehabilitation.

## The Development and Colonization of γδ T Cells

γδ T cells and αβ T cells originate from the same progenitor in the thymus. When bone marrow-derived hematopoietic stem cells (HSC) migrate into the thymus, Notch receptor 1 (Notch 1) and Delta-like 4 (DLL-4) signaling leads to the generation of T cell progenitors called double-negative cells expressing CD4^-^ and CD8^-^ (DNs, CD4^-^ and CD8^-^) (19, 21, 22), which commit them to the T-cell fate. Then these immature thymocytes pass through four developmental stages, from DN1 to DN4 (23, 24). DN1 cells are uniformly bipotent, they can give rise to both αβ and γδT cells (25); the next DN2 stage initiates the divergence of αβ and γδ T cells, and in this stage, cells expressing IL-7R and SOX13 (one high mobility group (HMG) box TF) and other unknown factors exhibiting the tendency to γδT cells fate (26, 27). TCR δ, γ and β start to rearrange stochastically (somatic recombination of the V, D, and J genes encoding the V domain of the corresponding TCR proteins) (28–30), and then weak signal strength boosts the divergence of αβ lineage (preTCR: consisting of the invariant pTαchain paired with a full-length β chain), while the strong signal enhances the γδT cells and selectively promotes the precisely rearranged and paired γδ chain (TCR γδ) (28, 29, 31–33), DETCs, IFN-γ-producing V γ1 cells and IL-17A-producing V γ6 cells are markedly depleted in mice with attenuated TCR signaling of their own (34, 35), this process is called the positive selection. The invalidly rearranged cells or validly rearranged cells without sufficient activation signaling from ligand undergo apoptosis similar to the death of the αβ T cells without useful TCR. Whether this phenomenon leads to the successive development characteristic of γδ T cells has to be verified. Partial cells of this stage retain bipotency, whereas other cells just give rise only to αβ or γδ T cells (36). The divergence of αβ and γδ lineage is completed at the DN3 stage, and by this stage, almost all of the cells complete lineage commitment, with a major population exhibiting αβ lineage restriction (25). But the precursor cells with type of TCR (preTCR or γδ TCR) can’t dictate the lineage choice, as the γδ TCR and αβ TCR can generate αβ and γδ lineage cells under some special circumstances, respectively (37–39); transitioning into the DN4 stage, the TCRα chain gene-rearrangement begins, which generates double positive(CD4^+^, CD8^+^) αβ T cells (DP αβ T cells) marking the point of irreversible commitment to the αβ lineage (36, 40). Then the DP αβ cells commit the positive and negative selection and get matured (41). While the subset of immature γδ T cells will develop the effector commitment, the relatively weaker signals enhance the IL-17–producing γδ T cell subset, and progressively stronger signals promote IFN-γ–producing and innate γδ T cells (24). However, there has no direct evidence whether the stronger or weaker signal leads to higher productions of IFN-γ- or IL-17A- V γ4 T cells, respectively. CD24 or heat-stable antigen (HSA) is recognized as the marker of γδ T cell lineage for irreversible commitment. The expression of CD24^+^CD73^+^ indicates that these cells are unable to switch to the αβ T cells (19, 42). Therefore, the TCR signaling operates in sequential developmental windows with distinct outcomes, and it determines the lineage and effector commitment successively (10). In addition, TCR γδ-independent factors are crucial in γδ T cells differentiation, such as the miRNAs, Sox4/Sox13/RORγ axis (SRY-box-containing gene 4/13/retinoid-related orphan receptor γ axis), and Notch signaling (13, 43, 44). Thus, every subset has its own development characteristic.

**Figure 1:**
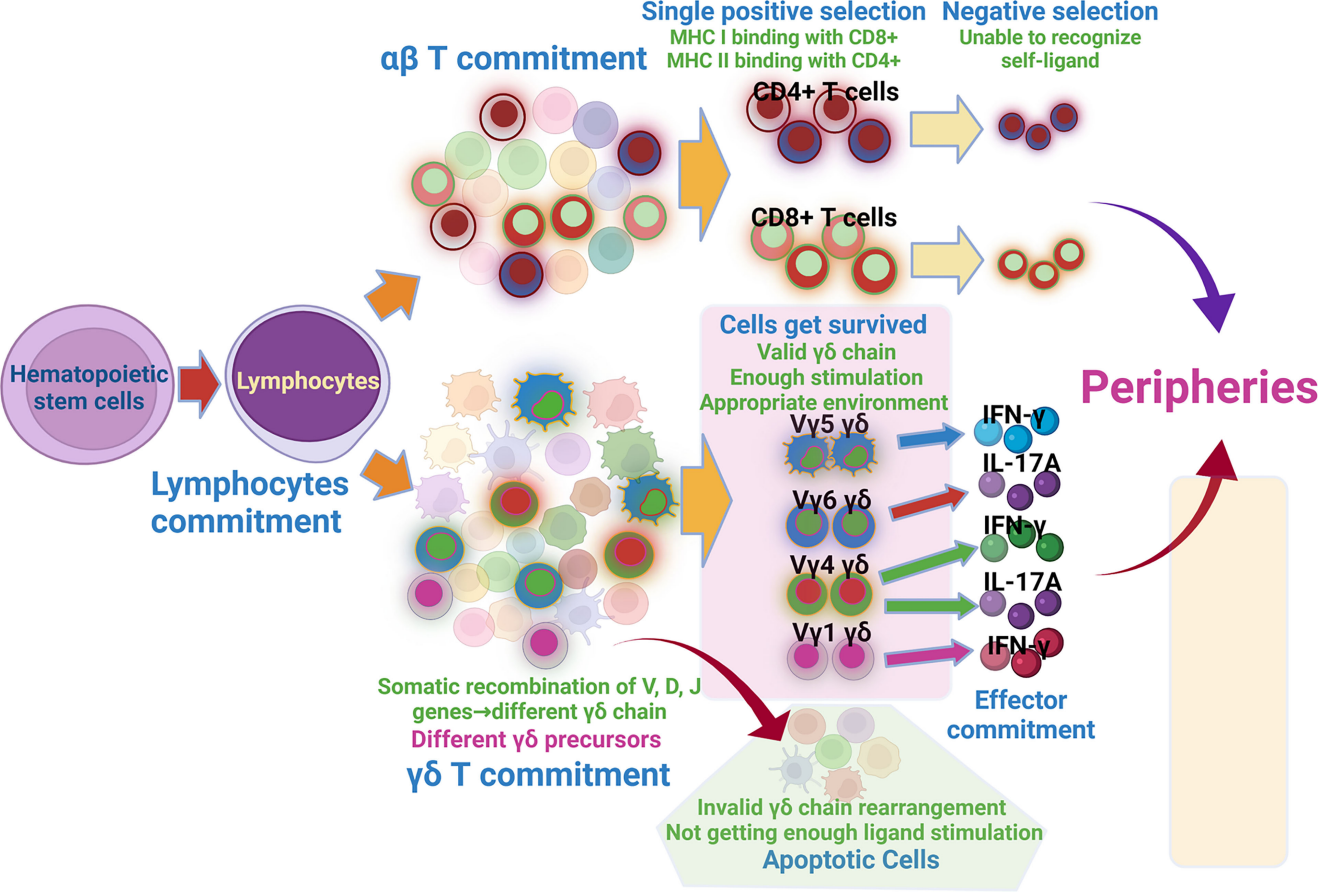
Development of αβ and γδ T cells. Hematopoietic stem cells migrating into thymus get lymphocytes commitment, the lymphocytes then get αβ commitment and γδ commitment. αβ cells passing through sequential single positive selection and negative selection get matured. Somatic recombination of V, D, J genes forms different γδ chain, which produces varied γδ precursors. Among them, cells with valid γδ chain, getting enough stimulation and appropriate environment get survived, cells with invalid γδ chain and getting insufficient ligand stimulation get apoptosis. Survived γδ T cells then undertake effector commitment and get matured.

The development of the γδ subset occurs step by step as follows: T cell commitment–αβ/γδ lineage commitment–γδ subset commitment–effector commitment (**Figure 1**); therefore, the same factor can take different functions during disparate stages. This theory can reconcile some inconsistent research results. For instance, IL-7 and the transcription factor SOX13 promote the survival and development of early precursor cells and are absolutely required for TCRγ gene rearrangement. However, at the later stage, their function mainly promotes the IL-17-producing cells (26, 27, 45, 46). Besides, the same factor can give rise to an identical or a different function for various subsets at the same cross-section in time, just like the PLZF and Egr2/3/id3; the former promotes the development of the Vγ1+ and Vγ6+ cells (47, 48), while the later one takes an opposite function in IL-17- and IFN-γ-producing cells (10).

DETCs expressing a canonical Vγ5Vδ1 TCR are a restricted antigen repertoire and act exclusively as resident T cells in the murine epidermis (12). They derive from DETC progenitors which are restrictedly generated in the embryonic thymus at day 13 to 17 (49), and at E16 and E18 (50), DETCs egress from the thymus and move to the epidermal layer where they self-renew. Existing research have confirmed that the development of DETCs can be influenced by ERK-Egr-Id3 axis (35), Lck (51), Syk (52), ZAP-70 (53), IL-7R/JAK/STAT pathway (54, 55), RunX3 (regulating CD103 and CD122) (56), miRNAs (downregulating CD122/IL-2Rβ and CD45RB expression) (43) and Skint-1 (promoting the selective development of Vγ5+ DETC) in the thymus (35, 57); their skin-homing are affected by the ITK (through promoting CCR10 and S1PR1 expression) (58, 59), SIPR1 (sphingosine-1-phosphate receptor 1, involved in thymic egress) (60), GPR15 (orphan G protein-linked chemoattractant receptor 15, regulating the recruitment of γδT cells to skin) (61), CD103 (62), E, P-selectins ligands (63) (Expressed on DETCs, binding to selectins expressed on the endothelium), CCR10 (64) and CCR4 (63) (binding to CCL27/28 expressed by keratinocytes), Vγ5 T cells have low expression in CCR9 and CCR7, so they will not migrate into lymphoid organ and spleen. Matured DETCs express the markers including CD27^–^, CD69^+^, T-bet^+^, NKG2D^+^, JAML^+^, CD100^+^, and CD103^+^ (15).

Vγ4 T cells appearing at the late fetal stage(from E16)and afterward (49), are the dominant subset of murine peripheral γδ cells. In addition, Vγ4 T cells exist in peripheral lymphoid organs, blood, liver, lung, spleen and dermis (65). They are divided into two main subsets: IL-17A^+^Vγ4 T cells (CCR6^+^CD27^-^), and IFN-γ^+^Vγ4 T cells (CCR6^-^CD27^+^) (66). The majority of γδ T cells in lymph node are IL-17A γδT cells, whereas a large population in splenic is IFN-γ γδ T cells (67); the mechanism leading to this biased distribution is unclear. The development of IL-17A producing cells is also regulated by the comprehensive factors, such as Sox4/Sox13/RORγt/IL-17 axis (68), Notch signaling/Hes-1 axis (44, 69), Wnt signaling pathway/TCF1 and Lef1 axis (70), TGF-β (71), Blk (B lymphoid kinase, a Src family kinase) (72) and IL-7 (45). Moreover, CCR6 is recognized to be critical for their homing to skin, CCR6-deficiency reduced the number of both Vγ4 and Vγ6+ cells in the skin (73). Other research reports that thymic Vγ4 requires extrathymic environment for skin homing, such as getting activated or obtaining CCR6 expression (74). Matured IL-17 producing Vγ4 T cells (thymus-derived) contain variable δ chain. Most of them express CD3+, CD4-, CD8-, CD44+, CD69+, RORγt+, CCR6+, CD25+, CD27-, Scart2+, CD45RB-, CD122-, CD27-, NK1.1-, T-bet-, IL-23R (31, 66, 75–80). Recent research found that some IL-17 producing γδ T cells are bone derived, and they often just have δ4 chain. In addition, they express CCR2+ and require IL-23 and IL-1β for their reprogramming from CD27+ γδ T cells (81, 82). In addition, IFN-γ-producing γδT cells are affected by ERK-Egr-Id3 axis (10, 34), ThPOK/PLZF/T-bet axis (83), researches have reported thymic γδ T cells with antigen-experience or binding antigen have high affinity in producing IFN-γ (67), matured IFN-γ producing Vγ4 T cells have variable δ chain. Their expression characteristics are CD3+, CD4-, CD8-, CD44+, T-bet+, NK1.1+, CCR6-, CD27+, CD45RB+, CD122+(IL-2/IL-15 receptor β chain) (31, 66, 75–80).

Vγ6 T cells, which exclusively express the Vδ1 TCR chain (74), are generated solely in the thymic second wave around embryonic day E14 (up to the birth) (49). In mice, about half of the dermal γδ T cells are the Vγ6 T cells, while the rest mainly express Vγ4 TCR (4, 74). Vγ6 T cells also localize to uterine epithelia, tongue and meninges, enthesis, pLNs, testis (79, 84–86). Conventionally, dermal Vγ6 T cells are considered bona fide tissue-resident cells that do not recirculate out of the skin and their generation is restricted to the confined window of fetal development. Furthermore, Vγ6 T cells cannot be induced in adult animals with the phenomenon that Vγ6+ γδ T cells become rare in the adult thymus (87, 88). But recent research confirmed that they have a high mobility and can travel between pLNs and tissues (79); however, whether the proliferated Vγ6+ in pLNs or thymus refill the pool of terminally differentiated skin Vγ6 remains to be tested. Their development is affected by IL-7 (45), TGF-β (71), Blk (72), PLZF (47). Matured Vγ6 cells exhibit the expression characteristics of CD27–, IL-23R+, RORγt+, CCR6+, CD69+, CD44+, Scart1+, cMAF+, PLZF+, PD-1 receptor and CCR2 (15, 79).

## γδ T Cells in Maintaining Skin Homeostasis

Skin comprises two major compartments, the epidermis and the dermis. The epidermis is mainly composed of keratinocytes (~95%) and residing immune cells (~5%, mainly are Langerhans cells (LC) and T cells) (89). The immune cell composition is subject to species specific differences. In naïve wild type (WT) mice, DETCs dominate the epidermal T cell compartment(~95%). Human epidermis is home to both γδ and αβ T cells, while resident T cells in epidermis show effector functions very similar to that of DETC (90).

The DETCs proliferate and maintain a homeostatic population by themselves, which cannot be reconstituted with bone marrow cells or fetal thymocytes (88). Aryl hydrocarbon receptor (AhR) and Linker for activation of T cells (LAT) are recognized to be the important factors in maintaining DETCs proliferative expansion and self-renewal (91). AHR-KO mice and LAT–deficient mice lack peripheral DETCs neither through affecting the DETCs generation nor skin homing (92). DETCs are characterized with lots of dendrites; most of the dendrites anchor to the apical epidermis where they are immobilized at distal. The remaining dendrites are positioned within the basal epidermis and are highly mobile (93). PALPs (containing prominent co-clusters of TCR and proteins phosphorylated on tyrosine residues) (94) of the apically oriented dendrites contribute the anchoring of DETCs to the squamous keratinocyte junctions, E-cadherin receptor integrin αEβ7(CD103) highly enriched at the ends of apical dendrites modulates the dendrite anchoring, which binds with E-cadherin expressed by keratinocytes. This structure allows the frequent contact of DETCs with the neighbouring cells as well as continuous scanning for antigens in the skin surface (94). Although healthy skin does not appear to express DETC TCR ligand detectable by soluble Vγ5Vδ1 TCR tetramers (95), low grade stresses from outside environment might sustain a basal expression of ligands sufficient for TCR activation but below the sensitivity of currently existed detection method. This presence of agonistic TCR-proximal signals make the DETCs to be a semi-activated state *via* Lck-dependent TCR activation (94), these semi-activated DETCs establish a polarized conduit system for transepithelial cargo transport, which contributes to the accumulation of matured lysosomes and the probe of the epidermal molecular composition (96). Normally, semi-activated DETCs express CD122 and CD69 (marker of pre-activation/semi-activation), their autocrine cytokines can help maintaining steady state of themselves and other cells (93), including IL-13, IGF-1, GM-CSF (**Table 1**). IL-13 plays an important role in regulating epithelial cells homeostasis and maintaining skin integrity through promoting EC (Epithelial cells) maturation and transiting through epidermis, the mice lacking canonical DETCs or IL-13 shows a higher degree of water loss, a poorer barrier function and a declined tolerance to damage compared to the WT skin (97); IGF-1 can protect themselves and keratinocytes from apoptosis (98), while GM-CSF is crucial for LC maturation (92). In turn, the paracrine cytokines by neighboring keratinocytes, fibroblasts and other cells are crucial in keeping the homeostasis of DETCs (96, 99). IL-7 secreted by keratinocytes and fibroblast mesenchymal cells serves as a growth factor for DETCs (100); IL-15 secreted by epithelial cells helps the survival and proliferation of DETCs *via* binding IL-15Ra (CD215) expressed on DETCs (101).

**Table 1:** Main cytokines, chemokines, and receptors of DETCs, Vγ4 and Vγ6 T cells in skin homeostasis and wound healing.

Cytokines	Main function	Receptors	Main function
IGF-1	Binding with IGF-1R, promotes keratinocytes survival and regulates their differentiation, prevents the apoptosis of DETCs.	CCR10/CCR4	Mediates DETCs migration and location *via* binding with CCL27/28.
KGF-1/KGF-2	Induces keratinocytes proliferation, differentiation and migration.	CCR6+	Contributes to homeostatic γδ T cells trafficking (Vγ4 and Vγ6).
IL-13	Regulates skin homeostasis and protects against carcinogenesis.	CCR2+	Dominates the trafficking of activated γδ T lymphocytes (Vγ4 and Vγ6).
GM-CSF	Is crucial for LC maturation.	αEβ7(CD103)	Contributes to the anchoring of DETCs.
IL-17A	Induces and amplifies inflammation, induces the migration of inflammatory cells.	AhR	Maintains DETCs proliferative expansion and self-renewal.
IFN-γ	Facilitates anti-tumor and anti-infection response.	IL-15Rα (CD215)	Maintains the survival and proliferation of DETCs and regulates the production of IGF-1 *via* binding with IL-15.
**Chemokines**	**Main function**	NCRs (NKG2D, TLR, CD100, JAML)	Provides costimulatory signals and participates in antigen recognition and inducing the release of cytokines.
CCL-3/CCL-4/CCL-5	Induces the migration of inflammatory cells.		
Mcp-1	Plays an important role in monocyte migration.		
XCL1	Induces migration of lymphocytes *via* binding with XCR1.		

The immune cells residing in the dermis under homeostasis include dermal subsets of dendritic cells (DCs), mast cells, T cells (αβ and γδ T cells), innate lymphoid cells (ILC), B cells, macrophages and NK cells (102). γδ T cells of dermis mainly comprised of Vγ4 and Vγ6 γδ T cells. Vγ6 γδ T cells represent virtually 100% of the dermal γδ T cells in newborn mice, but comprise only about 40% in adult mice, as the Vγ4 γδ T cells in the dermis gradually increase over time (103). The majority of Vγ6+ γδ T cells display tissue residency, but may retain the capability to circulate between tissues, while the Vγ4 T cells display the recirculating characteristic. Recent researches have indicated that both dermal Vγ4 and Vγ6 T subsets are radioresistant (74, 104). Under homeostasis conditions, both subsets can traffic between tissues and lymph nodes at a slow but steady rate (79, 87, 105, 106); a substantial flux of γδ T cells through the skin to draining LNs is observed through analysis of skin-draining lymph in cattle (107). It is proposed that CCR6-dependent manner contributes to homeostatic γδT17 cell trafficking, CCR6 can bind with CCL20 expressed in mucocutaneous sites and subcapsular region of primate LNs (108), while CCR2-dependent manner dominates the activated trafficking (73), this trafficking characteristic facilitates their immune surveillance function. Upon activated by ligands such as the specific ligands triggered by the imiquimod treatment, the migration will significantly increase. However, it seems that the Vγ4^+^ dermal cells are able to migrate more efficiently than the Vγ6+ γδT cells (103, 109). For the resident Vγ6γδT cells, they usually act as persistent effector cells in the skin, high expressions of the anti-apoptotic BCL2A1 protein protects them from activation-induced cell death (79). However, whether the resident Vγ6+ T cells can be refilled by the Vγ6 T cells from pLN and thymus is uncertain, and interesting to be tested. For the Vγ4 cells, they can be reconstituted by thymic Vγ4^+^ cells and bone marrow, but they need to go to the periphery and mature before migrating to the dermis (74, 81). The CCR6 expressed on their surface and the CCL20 expressed by epidermal keratinocytes, endothelial cells, and dendritic cells are crucial for their recruitment (82).

**Figure 2:**
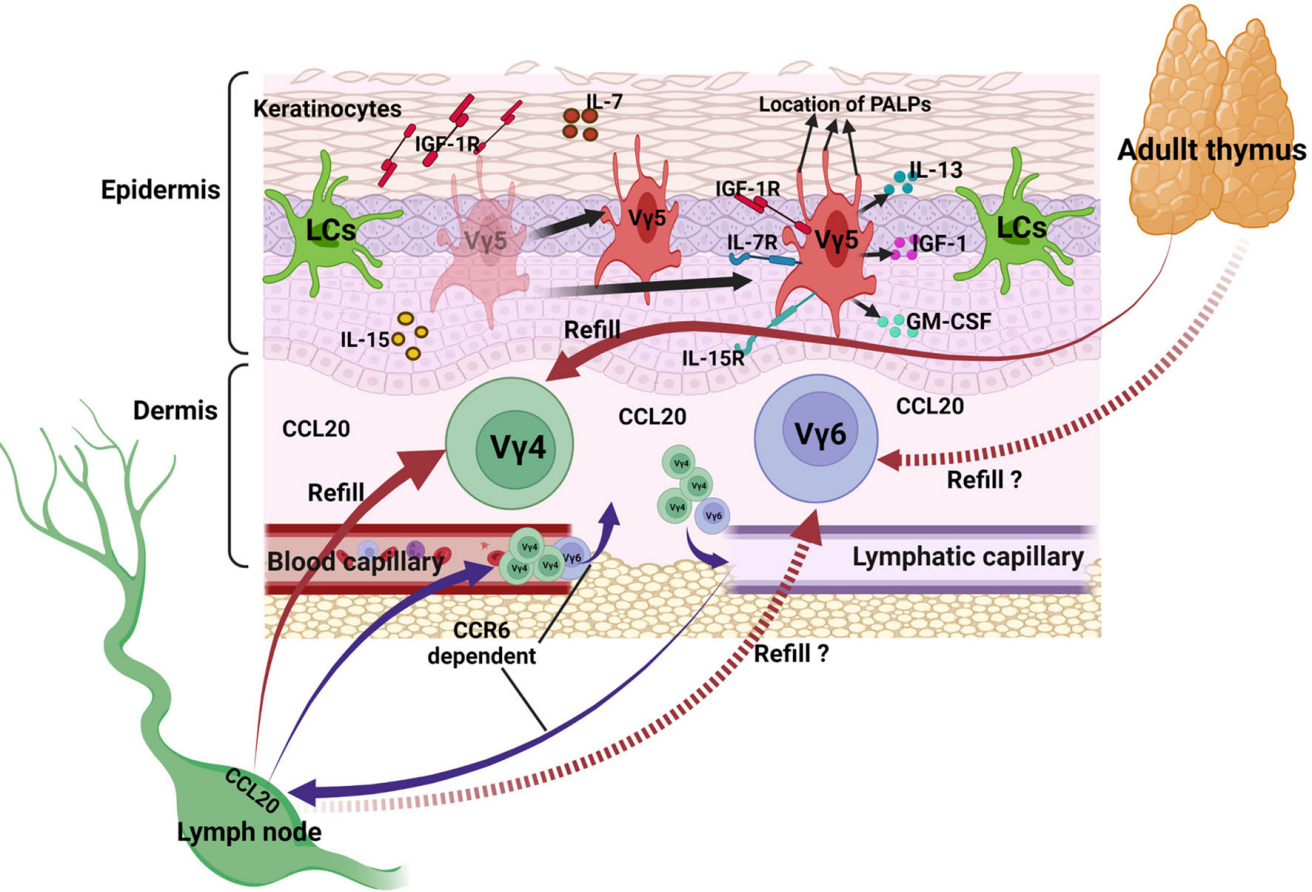
γδ T cells in maintaining skin homeostasis. DETCs in epidermis proliferate and maintain a homeostatic population by themselves, they secrete IL-13, IGF-1 and GM-CSF to help keeping steady state of themselves and other cells. IL-7 and IL-15 secreted by epithelial cells contribute to the survival and proliferation of DETCs, PALPs of the apically oriented dendrites contribute to the anchoring of DETCs to the keratinocyte junctions. Vγ4 and Vγ6 T subsets in the dermis traffic between tissues and lymph nodes at a slow but steady rate, CCR6 expressed on their surface combining with the CCL20 expressed in mucocutaneous sites and subcapsular region of primate LNs is an important pathway.

Collectively, DETCs exist in epidermis, they maintain a homeostatic population by self-renewal. Under homeostasis, they secrete IL-13, IGF-1 and GM-CSF to help in epithelial cells maturation and proliferation. IL-7 and IL-15 secreted by epithelial cells contribute to the survival and proliferation of DETCs, PALPs of the apically oriented dendrites contribute to the anchoring of DETCs to the keratinocyte junctions. Vγ4 and Vγ6 T are main subsets in the dermis, they traffic between tissues and lymph nodes at a slow but steady rate under homeostasis, CCR6 expressed on their surface combining with the CCL20 expressed in mucocutaneous sites and subcapsular region of primate LNs is an important pathway (**Figure 2**).

## The Activation of γδ T Cells

γδ TCRs have the ability for both innate and adaptive ligand recognition *via* either germline-encoded regions of the receptor, resemble the PRRs or adaptive antigen binding *via* the CDRs, this pattern seems to be distinguished from αβ TCRs (102). Most αβ TCRs bind to MHC I/II (major histocompatibility complexes I/II) which presents small peptide fragments derived from pathogens or pathological tissues. Together with co-receptor engagement of CD4 or CD8 and co-stimulation through CD28, this elicits αβ T-cell activation (110). Similar to αβ T cells, the activation of γδ T cells may require the engagement of both γδ TCR and co-receptors, including junctional adhesion molecule-like protein (JAML) (111), Toll-like receptor (TLR) (112), the semaphorin CD100 (113) and C-type lectin-like stimulatory receptor-natural killer group 2D (NKG2D) (114). As no general restricting molecule could be identified, no effective methods can assess whether the recognition of certain antigens by γδ TCRs is generalized, and the affinity of TCRs to their antigens is typically low, the antigens activating the γδ TCR or γδT cells have not yet been clearly identified up to now. Recent years, many studies have been conducted to explore the antigens. The antigens activating the γδ T cells can be divided into 4 categories (115): First of all, MHC or MHC-like recognition antigen includes MHC-Ib molecule T10/T22 (116), MART-1 (117), MHC-related protein 1 (MR-1) (118). Secondly, there are IG-like recognition of antigens, including Annexin A2 (119), ephrin receptor A2 (EphA2) (120), the human DNA mismatch repair protein MutS-Homologue 2 (hMSH2) (121), heat shock protein (HSP) 60 (122), PE(phycoerythrin) (123). Thirdly, this group contains Phosphoantigen, including 4-hydroxy-3-methyl-but-2- enylpyrophosphate (HMBPP), Isopentenyl pyrophosphate (IPP) and dimethylallyl pyrophosphate (DMAPP) (124). Lastly, there are B7 receptor family-like proteins, including BTNLs (BTNL1 and BTNL6 in mice, BTNL3 and 8 in human) (125, 126). Furthermore, the antigens can be categorized into DAMPs and PAMPs (damage associated molecular patterns and pathogen-associated molecular patterns) according to their derivation, the former ones are generated in cell necrosis (often associated with tissue injury), whereas the controlled cell death, or apoptosis, does not lead to the generation of DAMPs, the latter ones are elicited by pathogens (127). In addition, some papers divide the ligands into self ligands and non-self ligands (128).

Shortly after wounding or inflammation, damaged keratinocytes closely adjacent to the lesion quickly and transiently upregulate related stress antigen. The γδT cells of epidermis and dermis get complete activation *via* recognizing the antigens by TCR and co-stimulatory receptors. Activated epidermal γδT cells retract their dendrites and round up within 24 h after wounding (129). Within 48 h, epidermal γδ T cells secrete cytokines and growth factors to regulate inflammation and proliferation, such as KGF-1, KGF-2, IL-13, IFN-γ, TNF-α, IGF-1, IL-2, and IL-17 (**Table 1**), epidermal γδ T cells restore their dendritic morphology 5 days post wounding (4, 129). For the Vγ4 T cells, they are most commonly found early post wounding, accounting for half of the IL-17A^+^ cells on the third day (130), firstly, they get activated, proliferate and secrete IL-17A, IFN-γ, IL-17F, IL-22 and other cytokines to regulate the inflammation promptly. Secondly, the keratinocytes close to the lesion upregulate the production of CCL20, which increases the epidermal infiltration of dermal γδ T cells by binding their CCR6 (130, 131), in the absence of CCR6, fewer γδ T cells is observed at the wound site leading to 4-day delay in wound closure, this indicates a key role for CCR6 in efficient wound repair (132). The CCL20–CCR6 axis of dermal T cell recruitment occurs similarly in the human epidermis, resulting in Th17 cell infiltration (133). Thirdly, the migration of resident γδ T cells into the local draining lymph nodes increases, the traffic manner is CCR7-independent (105), and Vγ4^+^ cells homing from inflamed skin to sLNs during psoriasis predominantly lack CCR6 expression (109). It likely occurs *via* afferent lymph draining from dermis, but the definite pathway involved is undetermined. Fourthly, the γδ T cells specific expressing Vγ4Vδ4 in lymph nodes selectively expand promptly (105, 109), the reason leading to the selective expansion is uncertain, cytokines may play a crucial role in this process. Lastly, general γδ T cells and expanded Vγ4Vδ4 γδ T cells infiltrate back into inflammatory skin *via* S1P1 and CCR2 (82, 134), however, whether CCR2 up-regulation promotes the recruitment of thymus-derived Vγ4 T cells to inflamed tissue is unclear. Importantly, the re-filtrated Vγ4 Vδ4 T cells persist for months and respond more rapidly like the memory-like cells in the imiquimod (IMQ)-induced mice model (82). Activated Vγ6 T cells show very similar traits with Vγ4 T cells, CCR2 and CCR6 expressed on their surface are also crucial for the migration in homeostasis and inflammation state (73); however, it seems like their efficiency is lower than the Vγ4 cells (135).

**Figure 3:**
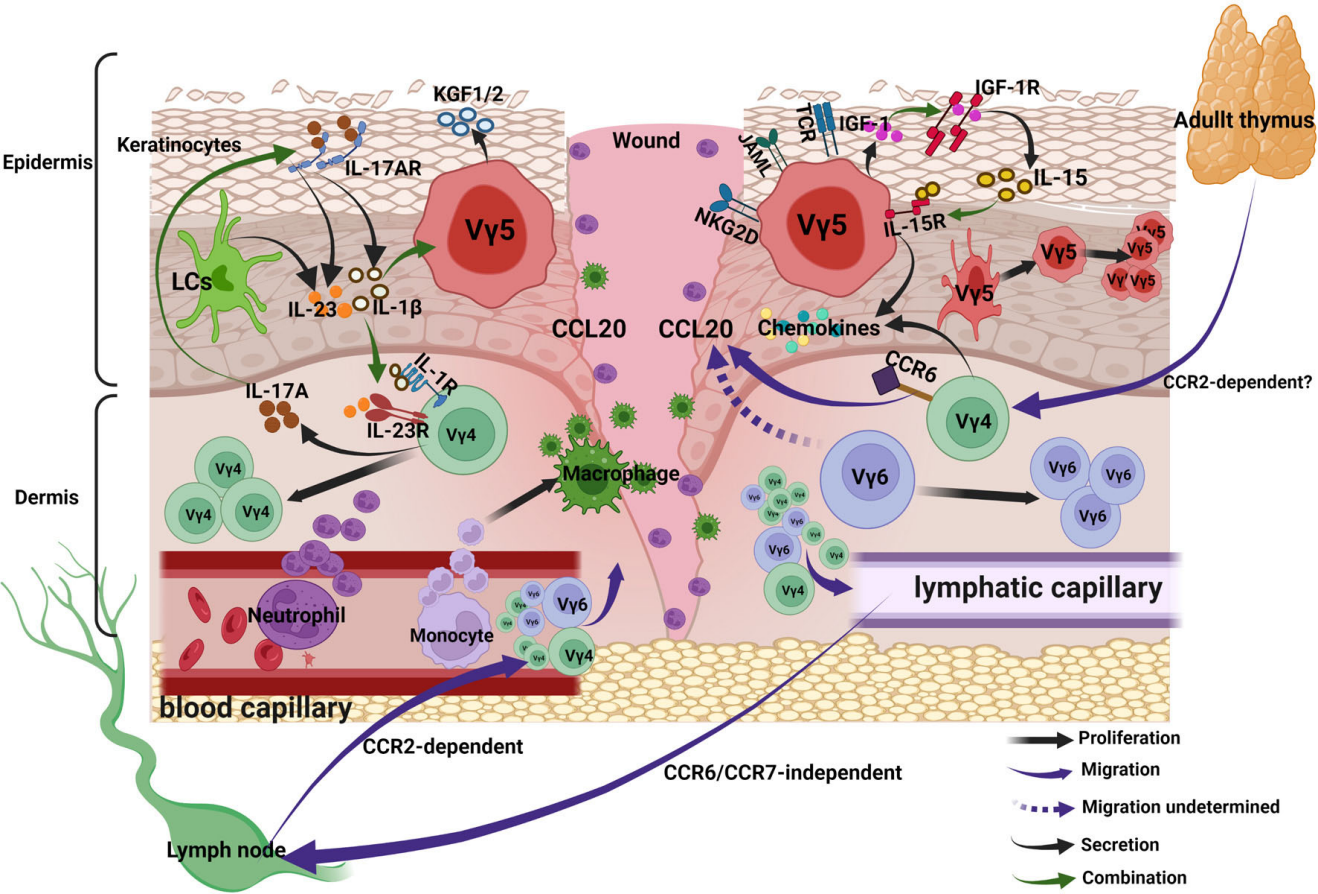
γδ T cells in acute wound healing. Upon activation, DETCs and Vγ4 T cells secrete chemokines to recruit neutrophils and macrophages into lesion site. Activated Vγ4 T cells migrate to epidermis *via* CCR6-CCL20 pathway, in addition, the traffic of Vγ4 and Vγ6 T subsets between skin and lymph nodes increases, the traffic from skin to lymph nodes is CCR6/CCR7-independent, while that from lymph nodes to skin is CCR2-dependent. Keratinocytes-derived IL-15 and DETCs-derived IGF-1 forms a positive feedback loop and promotes re-epithelialization. The positive feedback loop between wound-derived IL-1β/IL-23 and Vγ4-derived IL-17 can amplify the local inflammation, the IL-1β/IL-23 suppresses IGF-1 production of DETCs.

Taken together, the antigens activating the γδ T cells can be divided into 4 categories: MHC-like recognition antigens, IG-like recognition of antigen, phosphoantigen and B7 receptor family-like proteins; they can also be categorized into DAMPs and PAMPs. The binding of these antigens with the γδ TCR and co-stimulatory receptors helps in the complete activation of γδ T cells. Activated γδ T cells secrete chemokines, cytokines and growth factors to regulate inflammation and proliferation. Activated Vγ4 T cells migrate to epidermis *via* CCR6-CCL20 pathway, in addition, the traffic of Vγ4 and Vγ6 T subsets between skin and lymph nodes increases, the traffic from skin to lymph nodes is CCR6/CCR7-independent, while that from lymph nodes to skin is CCR2-dependent (**Figure 3**).

## γδ T Cells in Acute Wound Healing

The skin, the largest organ by surface area is susceptible to injury in shielding our internal tissues from microbial infection, temperature variation, radiation and mechanical damage (136). Recognizing the mechanism underlining the wound healing is valuable for regulating the healing effectiveness. Theoretically, both cells residing in skin and cells capable of trafficking to the skin as the keratinocytes, neutrophils, macrophages, T lymphocytes, mast cells, dendritic cells, endothelial cells, fibroblasts, myofibroblasts and epidermal stem cells, can influence the healing result (137–139). To observe their functions, a great number of surgically constructed models of skin injury in rodents have been established. In particular, murine models are used most often. It is well-established that appropriate inflammation and vigorous re-epithelization are crucial in wound healing, immune cells are essential in constructing inflammatory microenvironment and regulating re-epithelization (140). γδT cells as the major immune cells of skin, we sought to discuss their significant functions, and the related mechanism in wound healing below.

### Recruitment of Inflammatory Cells

Efficient Infiltration of inflammatory cells including neutrophils and macrophages are crucial for wound repair. Neutrophils are usually recruited as “first responders” from the bone marrow in response to “find me” signals on the day following injury, they clean debris and bacteria to provide a good environment for wound healing, as well as to modulate inflammation by producing ROS, chemokines (CXCL2, CXCL8) and MCP-1 (monocyte chemoattractant protein 1), different cytokines (IL-6, IL-1β, IL-10) (141). The accumulation of macrophages is usually seen within the 24-48 h at the site of injury, and their local accumulation actively participates in all stages of wound healing, including facilitating phagocytosis of bacteria and damage tissue, determining the duration of inflammation and promoting keratinocyte migration and ECM synthesis (142). Studies have confirmed that depletion, deletion, or excessive infiltration of these cells can result in delayed wound healing, keloids or hypertrophic scars (137, 143–146). γδ T cells participate in the recruitment of inflammatory cells in skin wounding. γδ TCR-deficient (δTCR^-^/^-^) C57 male mice exhibit reduction in the cellular infiltration upon injury, including macrophages, αβ T lymphocytes, neutrophils (104, 147, 148). Activated γδ T cells, including DETCs and Vγ4 T cells express CCL-3 (MIP-1α), CCL-4 (MIP-1β), CCL5 (Rantes), MCP-1, and XCL1 (lymphocyte chemokines), IL-17, which induce the migration of inflammatory cells (19, 106, 149–152). In addition, they indirectly affect cells infiltration *via* regulating other cells, such as DETCs-induced hyaluronan production by epithelial cells increases the migration of macrophages (153).

### Wound-Derived IL-1β/IL-23 and Vγ4-Derived IL-17 Loop for Inflammatory Responses

As the first line of defense, keratinocytes can recognize ligand by pattern-recognition receptors (PRRs) (154), which lead to the subsequent activation of distinct signaling pathways and the production of different cytokines and chemokines (138). TLR (Toll-like receptor) activation is a critical element in initiating and amplifying inflammation after skin injury, including TLR-1, -2, -3, -4, -5, -6, and -9, which are upregulated in wounds (155), The activation of keratinocytes increases the production of IL-1β, IL-23, IL-15, IL-1α, TNF-α, IL-8, CCL2 (156). Together with the IL-1β produced by platelets, neutrophils and macrophages (157, 158), as well as the IL-23 produced by LCs and DCs (159), the IL-23 and IL-1β induce the resident and infiltrated Vγ4 T cells secreting IL-17A (160, 161), which can bind with the up-regulated IL-17RA expressed on the keratinocytes. The binding enhances the production of epidermal IL-1β and IL-23 (130). Thus, this process creates a positive feedback that the IL-1β/IL-23-IL-17 loop amplifies local inflammation after skin injury. IL-17A, mainly produced by the immune cells, including γδT cells and Th17 cells, is required for efficient skin wound healing. IL-17a^-^/^-^ mice exhibit defects in wound repair (3); however, Rodero et al. reported that blocking IL-17A with an IL-17A-neutralizing antibody significantly promotes skin wound repair (162). To reconcile this conflicting result, Li et al. confirmed that different IL-17A levels play a distinct role in wound healing; both low and excessive levels of IL-17A have a negative impact on skin wound repair, while a moderate level of IL-17A is required for efficient skin wound healing (130). They concluded that Vγ4-derived IL-17A indirectly delayed the wound healing through upregulating of IL-1β and IL-23 by keratinocytes, which inhibits IGF-1 production by DETCs through NF-κB signal pathway (130). However, the underlining reason of different levels of IL-17A leading to variant effectiveness was not distinctly explicated in their study.

As we all know, IL-17A participates in inflammation through different pathways (163), we propose that the IL-17A—IL-1β/IL-23—IGF pathway impedes wound healing; whereas the IL-17A—β-defensin3/S100A8/Reg3γ/AMP (3, 164) and other pathways [through driving the production of VEGF by epithelial and fibroblastic cells to stimulate angiogenesis (165, 166)] promote wound healing. Under an excessive expression, the impeding pathway is markedly activated; therefore, IL-17A hinders the wound repair. Similarly, in the IL-17A-depleted mice, the promoting pathway is severely retarded, thus the wound healing is delayed. However, under a moderate expression, the promoting pathway is noticeably activated, IL-17A hence accelerates wound healing. It is worthy to explore these related molecular mechanisms for the details.

Moreover, we deliberate that these dual roles coexist at the same time, depending on the concentration gradient between the central injury tissue and the surrounding wounding tissue, reminiscent of the oxygen gradient in the wounding site (167). Moderate accumulation of IL-17A in the peripheries is beneficial for wound closure; while excessive accumulation of IL-17A at the excessive level in the center of injury leads to delayed repair, which leaves adequate time for inflammatory cells to create a good repair microenvironment. This process confirms the sequential order in repair, from the bottoms up and from the peripheries to the center (168). Further research is needed to justify this inference.

### DETCs-Derived IGF-1 and KGF-1-2 for Re-Epithelialization

During homeostasis, DETCs constitutively generate IGF-1, which binds to IGF-1R (IGF-1 receptor) expressed on “keratinocytes and DETCs” and triggers phosphoinositide 3-kinase and mitogen-activated protein kinase pathways to prevent them from apoptosis (98, 169). Meanwhile, keratinocytes secrete IL-15, which helps the survival and proliferation of DETCs (170). Upon injury, the production of IL-15 is upregulated by activated keratinocytes and Langerhans cells (170, 171), increased IL-15 enhances the IGF-1 production of DETCs through binding to their IL-15R (IL-15 receptor). The up-regulated IGF-1 causes an increase in phosphorylated IGF-1R levels at wound margins 24 h after injury (98). This in addition protects keratinocytes from apoptosis in damaged areas (98), also directly stimulates keratinocytes to produce more IL-15, partly through the mTOR-dependent pathway (172). This positive feedback loop of keratinocytes-derived IL-15 and DETCs-derived IGF-1 contributes to the significant accumulation of IGF-1, which exhibits a significant function in promoting re-epithelialization. Impaired epidermal to DETCs signaling slows wound repair (173), and it has been found that the insufficient activation of DETCs upon injury leads to abnormal wound healing in diabetic mice, the insufficient activation partly attributes to the impaired production of IGF-1. Exogenous supplement of IL-15 can rescue the defective IGF-1 expression (93). Whether there is another feedback loop between DETCs and other cells such as LCs, or other signaling deeply involved in the regulation of IL-15 expression is still unknown.

In addition to IGF-1, activated DETCs aid in skin repair by secreting KGF within 24 hours of injury, including KGF-1 and KGF-2 (174). However, they don’t secrete KGFs under homeostasis (129). When binding to the KGF receptor (KGFR) expressed on keratinocytes, KGF accelerates the migration and proliferation of keratinocytes by activating the downstream signaling pathways, including mTOR, ERK-MAPK, P13K/Akt (87, 96). KGF plays a commendable function in regulating keratinocytes, but since DETCs do not express KGFR, no positive feedback loop has been identified.

Taken together, upon activation, DETCs and Vγ4 T cells secrete chemokines to recruit neutrophils and macrophages into lesion site. Keratinocytes-derived IL-15 and DETCs-derived IGF-1 forms a positive feedback loop and promotes re-epithelialization. The positive feedback loop between wound-derived IL-1β/IL-23 and Vγ4-derived IL-17 can amplify the local inflammation, whereas the IL-1β/IL-23 suppresses IGF-1 production of DETCs (**Figure 3**).

## γδ T Cells in Chronic Wound Healing

**Figure 4:**
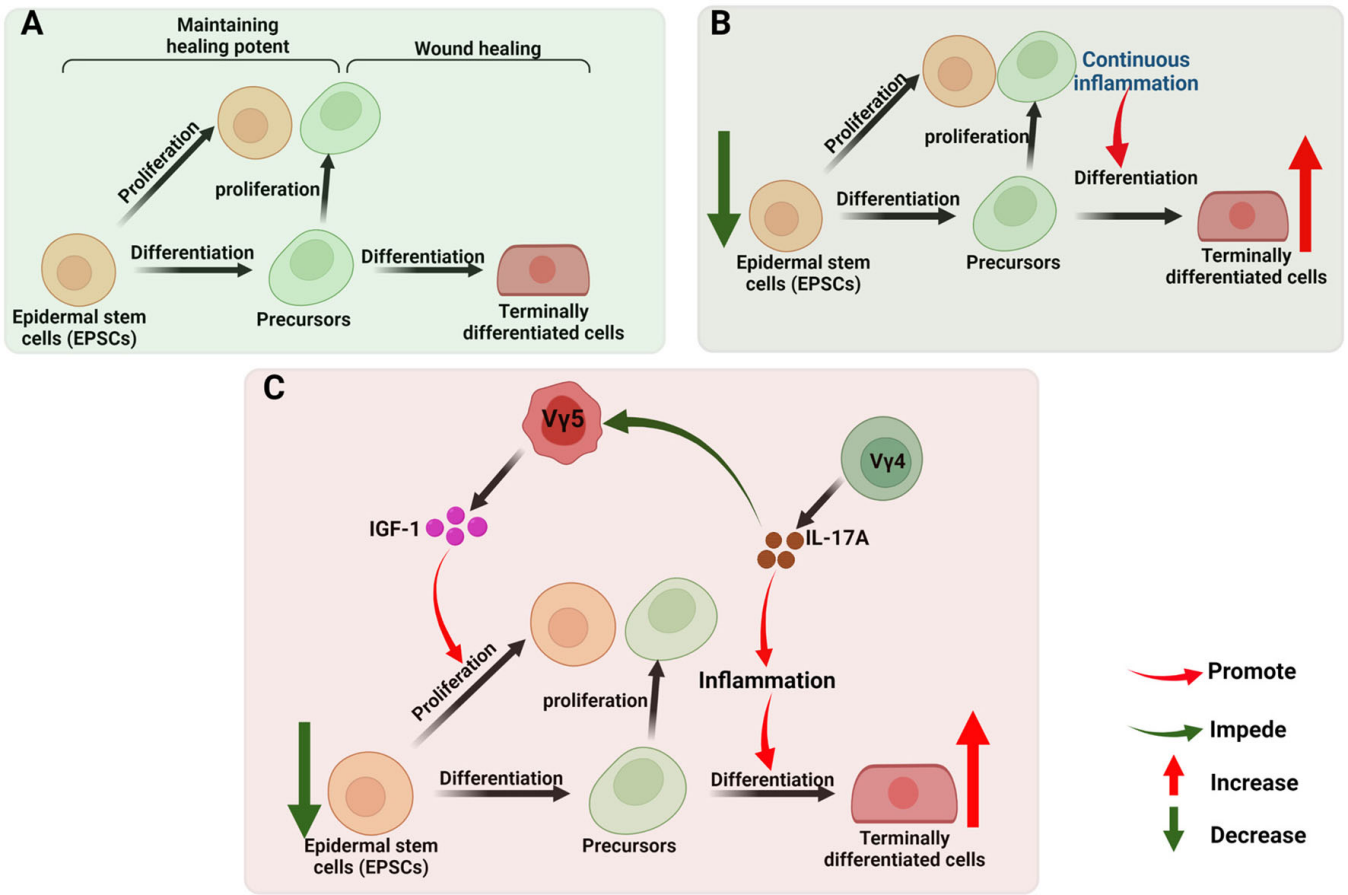
γδ T cells in chronic wound healing. **(A)** The robust activation of EPSCs and efficient recruitment of their progeny towards an epidermal lineage are crucial in the re-establishment of an intact keratinocyte layer during wound healing. The balance of proliferation of pluripotent EPSCs (maintaining healing potent) and their differentiation into terminally differentiated cells (wound healing) are pivotal; **(B)** In chronic or refractory wound, persistent inflammatory condition leads to excessive proliferation and differentiation, with the sacrifice of subsequent loss of the stem cell reservoir. **(C)** In chronic or refractory wound, continuous secretion of IL-17A by Vγ4 leads sustained inflammation which promotes the excessive differentiation, while suppresses the level of IGF-1 produced by DETCs beneficial for the proliferation of EPSCs, this inference is worthy to be tested.

Common features of chronic non-healing wounds include repeated infection, tissue necrosis, continuous exudation, defective re-epithelization, reduced angiogenesis and overproduction of ROS (175, 176). They are usually observed in elderly people suffering from pathological conditions, like obesity, diabetes mellitus and vascular disease (177). Chronic wound healing is characterized by the prolonged presence of myeloid cell populations, such as macrophages, neutrophils and monocytes. In the late stage of inflammation (137), incessantly activated γδ T cells participate in the chronic wound healing through inducing persistent inflammatory microenvironment *via* the main pathways mentioned above. For re-epithelialization, the robust activation of EPSCs (Epidermal stem cells) and efficient recruitment of their progeny towards an epidermal lineage are crucial, a stage which facilitates the re-establishment of an intact keratinocyte layer during wound healing (178, 179). For this process, the balance of proliferation of pluripotent EPSCs and their differentiation into terminally differentiated cells are pivotal (**Figure 4A**) (168, 180). In chronic or refractory wound, persistent inflammatory condition leads to excessive proliferation and differentiation, with the sacrifice of subsequent loss of the stem cell reservoir (181–183) and the balance is broken (**Figure 4B**). Supplementing sufficient EPSCs for restoring balance is the effective method to accelerate the wound healing (184–186). Our previous study found that DETCs-derived IGF-1 promotes the proliferation of EPSCs (187), while the IGF-1 secretion is regulated by Vγ4-derived IL-17A (130). So, we therefore hypothesize that the γδ T cells participate in regulating the differentiation and proliferation balance of EPSCs in refractory wound, the potential mechanism seems to be the continuous secretion of IL-17A by Vdifleads sustained inflammation which promotes the excessive differentiation, while suppresses the level of IGF-1 produced by DETCs beneficial for the proliferation of EPSCs (**Figure 4C**). Further research needs to be conducted in this regard.

Collectively, the differentiation and proliferation balance of EPSCs is crucial in wound healing, disordered immune microenvironment constructed by lymphcytes will break this balance in chronic and refractory wound. Given that the isolation and ex vivo expansion of various γδ T cell subsets is feasible (188), upon the molecular and cellular interations between γδ T cells and EPSCs being elucidated, precisely supplementing or clearing certain γδ T cell subsets, cytokines or chemokines in local will be an effective method to restore balanced microenvironment, which is expected to improve the effectiveness of clinical treatments for refractory wounds.

## Role of γδ T Cells in Other Skin Diseases

Fibrosis is essential for wound healing and tissue repair, which is characterized by the accumulation of extracellular matrix (ECM) components mainly produced by myofibroblasts. T lymphocytes, macrophages and other inflammation cells cooperatively regulate fibrotic process (189).

Studies have found γδ T cells play critical roles in fibrosis and fibrotic diseases of many tissues, including hepatic, lung, kidney and heart. IL-17/IL-22 producing γδ T cells can protect the liver from excessive fibrosis *via* inducing HSCs (hepatic stellate cells) apoptosis (190). Besides, IFNγ-producing γδ T cells also show protective effect in liver fibrosis, these cells have direct cytotoxicity against activated HSCs (191). For lung, Vγ6Vδ1 γδ T cells protect it from pulmonary fibrosis by secreting IL-22 (192). However, some researches demonstrate γδ T cells accumulation tends to promote fibrosis, IL-17-producing γδ T cells induces myofibroblast activation and ECM deposition in kidney injury model and myocardial infarction model of mice (193, 194). So, it is more likely that their function in regulating fibrosis is tissue-specific.

Up to now, researches related to the γδ T cells in skin fibrosis is inadequate, Ohtsuka found the human skin fibroblasts stimulated by γδ T cells supernatant showed elevated proliferation and collagen synthesis (195), another study demonstrated the activated γδ T cells in systemic sclerosis (SSc) play an important role on fibrosis (196). In addition, Meyer demonstrated epidermal γδ T cells induces profibrotic response of fibroblasts *via* mice in chronic inflammation, this phenotype of mice lacking fibroblast growth factor bears continuous inflammatory response (197). Recently, Shook (198) found CD301b-expressing macrophages activated the proliferation of wound bed adipocyte precursors (APs) through IGF-1, these Aps become fibrotic after injury. DETCs secreted sufficient IGF-1 upon skin injury, whether they can play equivalent effect deserves further study.

For immune-mediated skin diseases, psoriasis, atopic dermatitis (AD) and contact dermatitis (CD) are all chronic and prevalent (15). The prevalence of psoriasis is about 2% to 3% (199), γδ17 T cells have been proved to be critical in imiquimod- (IMQ) or IL-23-induced psoriasis of mice, both Vγ6 and Vγ4 are clearly pathogenic in these models (131), memory-like dermal Vγ4 γδ17 T cells accumulated in inflamed skin and peripheral lymph nodes lead to faster and stronger responses upon secondary challenge (82). STAT 3 and STAT 4 facilitate the complete effector functions of γδ17 T cells (200). PD-1 and CD109 exert protective role in psoriasis (201, 202), while LAT1 and CD69 exert opposite function (203). In humans, patients with psoriasis also display increased accumulation of γδ T cells (Vγ9Vδ2) in the skin, effective therapy can decrease the numbers, indicating their role in the disease (204). AD is a T cell-mediated chronic skin disease, affecting up to 20% of children worldwide, its onset is associated with skin barrier dysfunction and immune disorder (205), it is characterized by highly expanded dermal αβ T cells which produce IL-17 and IL-22 (206), patients suffered from AD also present decreased proportion of γδ T cells (207). However, children with AD display higher frequency of Vγ9Vδ2 T cells (208). So the specific role and underlined mechanism of γδ T cells in AD is worthy to investigate. CD is the most frequent immune-mediated skin disease, its prevalence is about 95%, which is caused by chemical and allergens (209). The role of DETCs in CD is controversial (15), IL-17 secreted by Vn CD is controversialsed by chemicalproinflammatory role (106), however, their respective role in CD needs to be evaluated in depth.

## Discussion and Conclusion

γδ T cells are important components of the skin immune system and DETCs(Vγ5), Vγ4 and Vγ6 T cells are their major subsets. DETCs are particularly generated in the embryonic thymus and implanted in the epidermis where they maintain a homeostatic population by themselves. Vγ4 T cells appearing in the late fetal stage can be generated in the adult thymus, and they possess the recirculating characteristic which can be refilled by newly generated Vγ4 cells from thymus and pLN. Vγ6 T cells are generated solely in the thymic second wave around embryonic day E14 (up to the birth), and they mainly display tissue residency, but retain circulating capability, whether they can be refilled by circulating cells is uncertain. The development and differentiation of γδ T cells are regulated by both TCRγδ-dependent and TCRγδ-independent factor. The combined effect of various factors leads to the differentiation of γδ T cells. Their functional development is accomplished step by step as follows: T cell commitment–αβ/γδ lineage commitment–γδ subset commitment–effector commitment.

Under homeostasis, γδT cells participate in maintaining skin integrity with the help of paracrine and autocrine factors, traffiking between tissues and lymph nodes of Vγ4 and Vγ6 T cells at a slow rate in the steady state which plays an important role in immune surveillance. Besides, these cells are radioresistant, for mice receiving lethal irradiation, 100% of DETCs (V0%+) remained of host origin, while 90% of Vγ5-γδ T cells in dermal remained host-derived (104). Upon injury or inflammation, antigens including MHC-like recognition antigens, IG-like recognition of antigen, Phosphoantigen or B7 receptor family-like proteins are upregulated. The binding of these antigens with the γδTCR and co-stimulatory receptors helps in the complete activation of γδT cells. Initially, activated γδT cells secrete chemokines to recruit the inflammatory cells, including neutrophils and macrophages etc. Subsequently, they secrete IGF-1, KGF-1/KGF-2, IL-17 to regulate inflammation and re-epithelialization. Injury provide an opportunity for microorganisms to enter into the wound tissues, including microorganisms constituting the skin microbiota and residing in the environment.

It is noteworthy to mention that the positive feedback loop of DETCs-derived IGF-1 and keratinocytes-derived IL-15 leads to the accumulation of IGF-1 in wound bed, on one hand, it protects keratinocytes and epidermal γδ T cells from apoptosis, on the other hand, it exhibits a significant function in promoting re-epithelialization, γδ T cells in the epidermal of both mice and humans show equivalent function. In the dermal, the wound-derived IL-1β/IL-23 and Vγ4-derived IL-17 feedback loop can amplify the local inflammation. IL-17A participates in regulating wound healing by either promoting pathway (like the IL-17A—IL-1β/IL-23—IGF pathway) or impeding pathway (like the IL-17A—β-defensin3/S100A8/Reg3γ/AMP pathway). Different doses affect each pathway to different degrees, both low and excessive levels of IL-17A have a negative impact on skin wound repair, while a moderate level of IL-17A is required for efficient skin wound healing, suggesting that IL-17A plays a varied role in wound healing. For chronic and refractory wounds, they provide a lot of opportunities for microorganisms to enter into the wound tissues (210), including commensal microbiota residing in the skin and microorganisms existed in the environment, pathogenic interaction of microorganisms with the skin cells will induce pathogenic immune response (177, 211). In this process, abnormal accumulated γδ T cells or their disordered function contribute to unbalanced immune microenvironment, which breaks the differentiation and proliferation balance of EPSCs, restoring balanced microenvironment is expected to improve the effectiveness of clinical treatments for refractory wounds. Further research needs to be conducted in this regard.

In addition, γδ T cells play critical roles in fibrosis and fibrotic diseases of many tissues, their protective or deleterious function in fibrosis is more likely tissue-specific. Up to now, researches related to the γδ T cells in skin fibrosis is inadequate, investigating their role in keloids and hypertrophic scars forming is valuable. For immune-mediated skin diseases, both Vγ6 and Vγ4 are clearly pathogenic in imiquimod-induced psoriasis, their function in atopic dermatitis and contact dermatitis needs to be evaluated in depth.

## Author Contributions

WGH and RS wrote the manuscript. JY and CC participated in the project discussion. ZL made some valuable suggestions about manuscript structure. GPL helped to design the manuscript structure and edited the language. WFH and GXL evaluated and reviewed manuscript structure, ideas and science. All authors contributed to the article and approved the submitted version.

## Funding

This work was supported by funds from the National Natural Sciences Foundation of China (No. 31872742 and 82172232 to WFH, No. 81630055 and No. 81920108022 to GXL).

## Conflict of Interest

The authors declare that the research was conducted in the absence of any commercial or financial relationships that could be construed as a potential conflict of interest.

## Publisher’s Note

All claims expressed in this article are solely those of the authors and do not necessarily represent those of their affiliated organizations, or those of the publisher, the editors and the reviewers. Any product that may be evaluated in this article, or claim that may be made by its manufacturer, is not guaranteed or endorsed by the publisher.
